# A deep learning framework for threshold-free relative gene expression ranking from histone modification signals

**DOI:** 10.1186/s12859-026-06553-6

**Published:** 2026-07-23

**Authors:** Abdul Munif, Amitava Datta, Zhaoyu Li, Max Ward

**Affiliations:** 1 School of Physics, Mathematics, and Computer Sciences, The University of Western Australia, 35 Stirling Highway, Crawley, Western Australia 6009 Australia; 2 School of Biomedical Sciences, The University of Western Australia, 35 Stirling Highway, Crawley, Western Australia 6009 Australia

**Keywords:** Deep learning, Pairwise ranking, Histone modifications, Gene expression, Epigenetics

## Abstract

**Background:**

Current methods for predicting gene expression from histone modifications rely on arbitrary binary classification thresholds such as the median to distinguish between high and low expression for genes. This approach lacks biological justification, creates dataset-dependent classifications, and ignores the relative regulatory relationships between genes that are often more biologically meaningful than absolute cutoffs.

**Methods:**

We developed a deep learning method using pairwise ranking to determine the relative gene expression levels between gene pairs based on their histone modification signals. The approach adopted the DirectRanker architecture, which enforces antisymmetry by construction through a twin-subnet design with asymmetric subtraction, and was evaluated against three baseline classifiers (Random Forest, Logistic Regression, and SVM Linear) on identical features and data splits. Ablation testing across all 31 non-empty subsets of five histone marks (H3K4me3, H3K9ac, H3K9me3, H3K27ac, H3K27me3) was conducted to identify their individual contributions. Datasets were strictly partitioned (80% training, 10% validation, 10% testing) with gene-level separation to prevent data leakage, and all experiments were repeated across five independent random seeds. Four datasets were analysed: two normal adult liver cell datasets (Donor 3 and Donor 4 from NCBI GEO GSE19465) and two HepG2 hepatocellular carcinoma cell line datasets (ENCSR134DWG and GSE76344).

**Results:**

Multi-mark combinations anchored by active histone marks (H3K27ac, H3K4me3, H3K9ac) consistently drove predictive performance, with the best combinations achieving AUROC values of 0.833−0.867 and test accuracies of 74–78% across datasets. DirectRanker attained AUPRC values of 0.819−0.856, closely matching or exceeding Random Forest on precision–recall performance. Repressive marks (H3K9me3, H3K27me3) consistently underperformed when used in isolation, with single-mark models achieving test accuracies of only 50–66%. DirectRanker produced substantially higher antisymmetry scores (0.961−0.983) than all baseline classifiers (0.787−0.851), confirming logically consistent pairwise predictions. Performance plateaued at four- to five-mark combinations, reflecting correlated predictive signal among the active marks within the promoter-proximal feature space.

**Conclusions:**

The pairwise ranking framework provides a principled alternative to threshold-based classification, enabling relative gene expression comparisons without arbitrary binarisation cutoffs. While Random Forest achieved marginally higher accuracy, DirectRanker’s structural antisymmetry guarantee makes it the more suitable choice for applications requiring a globally coherent gene ranking. Active promoter marks carry the majority of the predictive signal, while the performance saturation with increasing mark combinations reflects overlapping information content rather than biological redundancy.

## Background

Epigenetics is a field that explores heritable changes in gene expression without alterations to the DNA sequence. It is increasingly significant due to its implications in various diseases, cancer, cognitive dysfunction, and autoimmune disorders [1]. These changes are influenced by environmental factors such as exposure to heavy metals, pesticides, and tobacco smoke, as well as nutritional elements, with the potential to persist across multiple generations. Epigenetic modifications occur through mechanisms like DNA methylation, chromatin modification, imprinting, and the involvement of non-coding RNAs, each playing a critical role in gene regulation and disease development [2, 3].

Recent advancements have enhanced our understanding of the complex molecular landscape of diseases like cancer. However, traditional methods often fall short in capturing the nonlinear relationships present in biological data. This limitation has led to the exploration of deep learning models, which offer the potential to uncover more complex patterns and correlations between histone modifications and gene expression. Models like DeepChrome [4] and its successor, DeepChrome 2.0 [5], utilize convolutional neural networks and generative adversarial networks (GANs) to improve prediction accuracy and provide deeper insights into the epigenetic regulation of genes.

The research proposes a novel approach to gene expression prediction, utilizing the pairwise ranking approach, where the relative expression levels of two genes are compared based on their histone marks. Based on learning-to-rank approaches [6–8], we aim to develop a neural network architecture for pairwise ranking. The primary objective is to identify which combinations of histone marks most strongly predict relative expression differences between gene pairs.

Deep learning is famous for its capacity to find complex relationships between the data. Singh et al. [4] gave a novel computational framework for predicting gene expression based on histone modification data. The framework, called DeepChrome, was built based on convolutional neural networks (CNNs). While DeepChrome showed competitive performance with traditional methods like Support Vector Machines and Random Forests, subsequent studies like ShallowChrome [9] have suggested that the performance gains of deep learning approaches in this domain may be more modest than initially reported.

[10] later developed an attention-based approach called AttentiveChrome. Attention was proposed to help AttentiveChrome identify relevant positions within chromatin markings. It consists of five modules which are Bin-level LSTM encoder for each histone modification (HM) mark, Bin-level Attention on each HM mark, HM-level LSTM encoder that encodes all HM marks, HM-level Attention over all the HM marks, and the classification module. The drawbacks of AttentiveChrome are that the results rely on correlation with only H3K27ac for validation, which is available for just 3 out of 56 cell types. The model also assumes a sequential ordering of histone marks even though no clear biological ordering exists.

[11] presents neural network layers with self-attention mechanism and transfer learning, called TransferChrome, to improve prediction accuracy, especially in across cell lines. The transformer architecture was used as self-attention mechanism for aggregating global information. While TransferChrome achieved marginally higher AUC scores than existing methods (0.043 improvement over DeepChrome, 0.023 over AttentiveChrome, and only 0.006 over ConvChrome), these gains are modest and may not justify the added architectural complexity. The arbitrary selection of E085 as the source domain for all cross-cell line experiments lacks justification and undermines the generalizability claims of the transfer learning approach.

[9] introduce ShallowChrome, a novel computational pipeline for the accurate and interpretable prediction of gene expression based on histone modifications. It uses a feature extraction process based on peak calling from ChIP-Seq data. This method identifies significant genomic regions where histone marks are enriched and uses these features for gene expression prediction in binary classification. Because ShallowChrome models use a logistic regression model, it can be interpreted straightforwardly from its model parameters. While this linear regression coefficient claimed to provide meaningful biological interpretability, these weights merely show statistical association rather than causal mechanism. The validation using only one gene (PAX5) across three cell types may not provide sufficient evidence about extracting regulatory insights across thousands of genes and dozens of epigenomes.

The main challenge encountered by the deep learning approach is interpretability [4, 9–11]. Deep learning is often seen as a black box model, making it hard to interpret the learned interactions and the effect of histone modifications on gene predictions. The inability to achieve accurate predictions across different cell lines has also been studied [11]. The usage of binning strategies, where separate models were trained for each bin of DNA regions, could not figure out the interactions between neighboring bins. This is important for understanding the spatial distribution of histone modifications. A more complex model can improve the model accuracy, but also increase the model interpretability. It is difficult to interpret the model’s predictions and understand the contribution of different histone modification marks to gene expression [9, 11].

## Related works

### Contrastive learning

Traditional supervised learning relies on large labeled datasets, which are costly and time-consuming to produce. On the other hand, contrastive learning learns effective representations from unlabeled data by training models to distinguish between similar and dissimilar samples [12]. It is widely used in image classification, object detection, video analysis, and natural language processing.

[13] introduced SimCLR, a framework for learning visual representations without human supervision. They applied a sequence of augmentations—random crop/resize, color distortion, and Gaussian blur—to generate two correlated views of each image. [14] proposed a Supervised Contrastive (SupCon) loss to address SimCLR’s limitations. SupCon uses multiple positives per anchor, two loss formulations, two-stage training, and normalized embeddings. It consistently improved performance on ResNet-50, ResNet-200, CIFAR-10, and CIFAR-100, and showed greater stability across hyperparameters. Its drawbacks include the need for large batch sizes, a separate pretraining and classification stage, higher computational cost, limited transfer-learning gains compared with cross-entropy, and evaluation restricted mainly to ResNet architectures.

In our setting, ground-truth expression labels are directly available for every gene, and the task is inherently relational where we want to determine which of two genes has higher expression. This makes a supervised pairwise ranking formulation more natural and sufficient than a contrastive representation learning approach. Rather than learning an embedding space, we directly exploit the gene expression level to construct binary-labeled gene pairs, making the full contrastive learning pipeline unnecessary.

### Pairwise ranking

[6] proposed a new approach called RankNet to improve the ranking accuracy and efficiency in search engines. The goal of their approach was to order a set of documents returned for a given query based on their relevance. The main components of the approach are probabilistic cost function based on pairs of examples, the use of neural networks to model the underlying ranking function to capture complex relationships in the data, and modified backpropagation algorithm for training on pairs of examples. This resulting ability to learn complex ranking function and improved performance with increasing training set size and handled ties effectively.

An additional method called LambdaRank and LambdaMART has been proposed to improve the search result quality, optimizing information retrieval measures (e.g. NDCG) [7]. LambdaRank builds upon RankNet and directly specifies gradients (lambdas) instead of deriving them from a cost function. The LambdaMART combines LambdaRank with MART (Multiple Additive Regression Tree). It uses gradient boosting with regression tree. These proposed methods can be easily adapted to optimize various IR (Information Retrieval) measures.

A pairwise learning-to-rank approach called DirectRanker was proposed by [8]. This approach generalizes the RankNet architecture, making it simpler. The main components of the algorithm lies in a neural network structure with two identical subnets that process document features, followed by taking their difference and passing it through an output neuron. The model is proven to be reflexive, antisymmetric, and transitive, allowing for simplified training and improved performance. These properties enable training on only neighboring relevance classes and avoid the need to explicitly train on document equality within classes.

## Methods

### Dataset

We use four datasets, consisting of two healthy liver datasets and two HepG2 liver cancer cell datasets. We selected these two types of datasets to test whether our method can be applied across different biological conditions and to examine potential differences in their behavior. The sources of the datasets are listed in Table 1. We use five histone marks in this experiment: H3K4me3, H3K9ac, H3K9me3, H3K27ac, and H3K27me3. These histone marks are available in all datasets, and their detailed descriptions are provided in Table 2.

**Table 1:** Dataset used in the experiments

Name	Description	Source
Donor ID 3	Homo sapiens liver tissue female adult (25 years)	[15]
Donor ID 4	Homo sapiens liver tissue male adult (31 years)	[16]
HepG2 GSE76344	Histone modification and nucleosome mapping in human liver cancer cells	[17]
HepG2 ENCSR134DWG	REMC Epigenome (ENCODE) for Hepatocellular Carcinoma Cell Line using donors/samples	[18]

**Table 2:** Histone marks used in the experiments

Histone mark	Associated with	Functional category
H3K4me3	Promoter regions	Promoter mark
H3K9ac	Promoter regions	Promoter mark
H3K27ac	Enhancer and promoter regions	Distal mark
H3K9me3	Heterochromatin regions	Repressor mark
H3K27me3	Polycomb repression	Repressor mark

### Dataset preprocessing

**Fig. 1:**
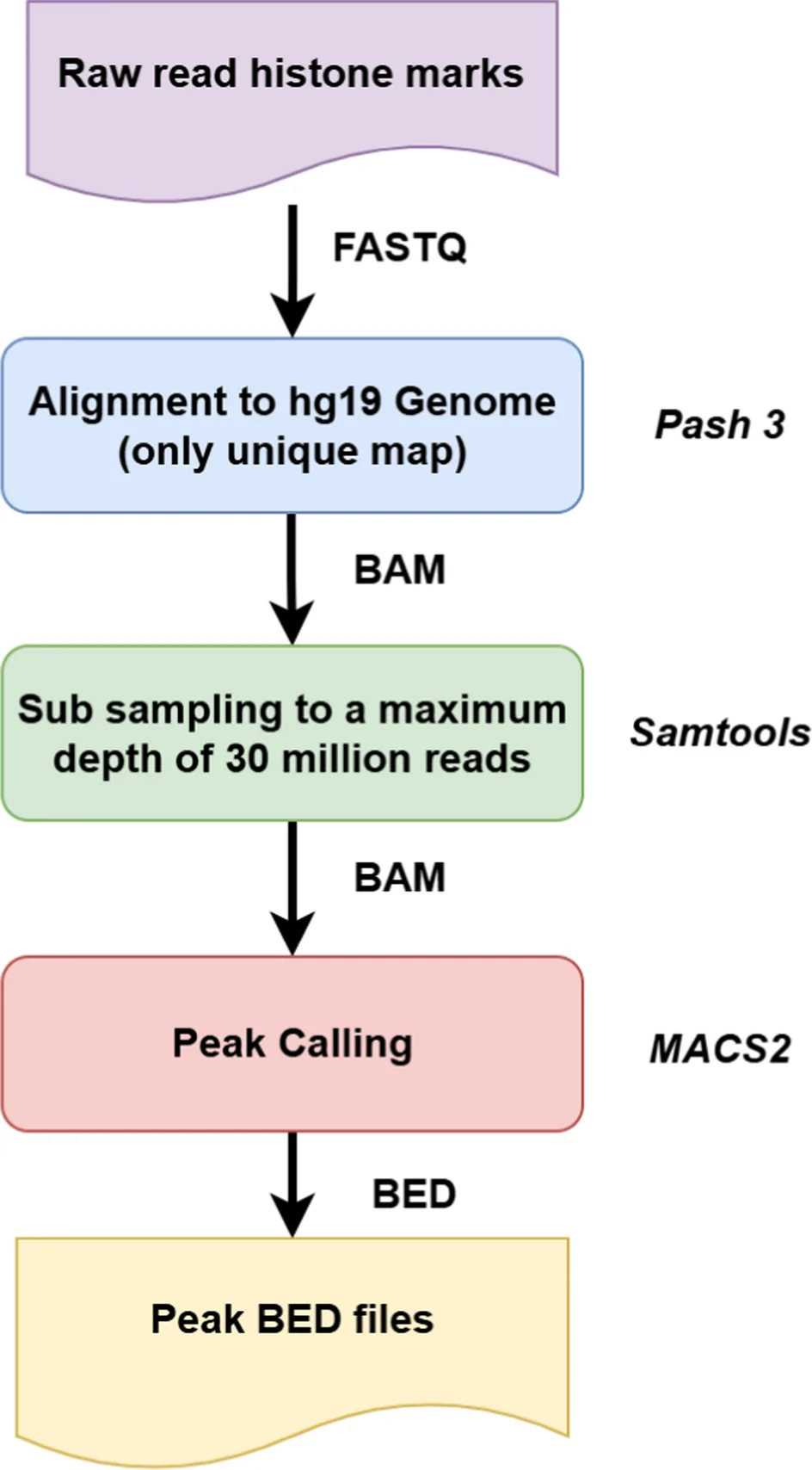
Dataset preprocessing

We followed the same preprocessing pipeline used by the Roadmap Epigenomics Project (REMC) for all datasets to obtain peak values [19]. The overall workflow is summarized in Fig. 1, and each step is explained below.

1. Read Mapping
  Raw histone mark reads in FASTQ format were mapped to the hg19 human genome assembly using the Pash 3.01.02 read mapper. Only uniquely mapped reads were retained, and duplicate reads were removed.
2. Subsampling
  All histone mark datasets were uniformly subsampled to a maximum depth of 30 million reads. These subsampled datasets were then used for subsequent peak calling.
3. Peak Calling
  Peaks were identified using MACS v2.2.9.1 by comparing each ChIP-seq dataset against its corresponding whole cell extract (WCE) control. Narrow peaks were detected using a Poisson p-value threshold of 0.01, and broad domains were identified using an additional broad-peak p-value threshold of 0.1. Fragment lengths were pre-estimated using strand cross-correlation analysis from the SPP peak caller package [20], and these estimates were explicitly passed to MACS2 via the –shift-size=fragment_length/2 parameter.

### Proposed method

#### Pairwise ranking using deep learning

**Fig. 2:**
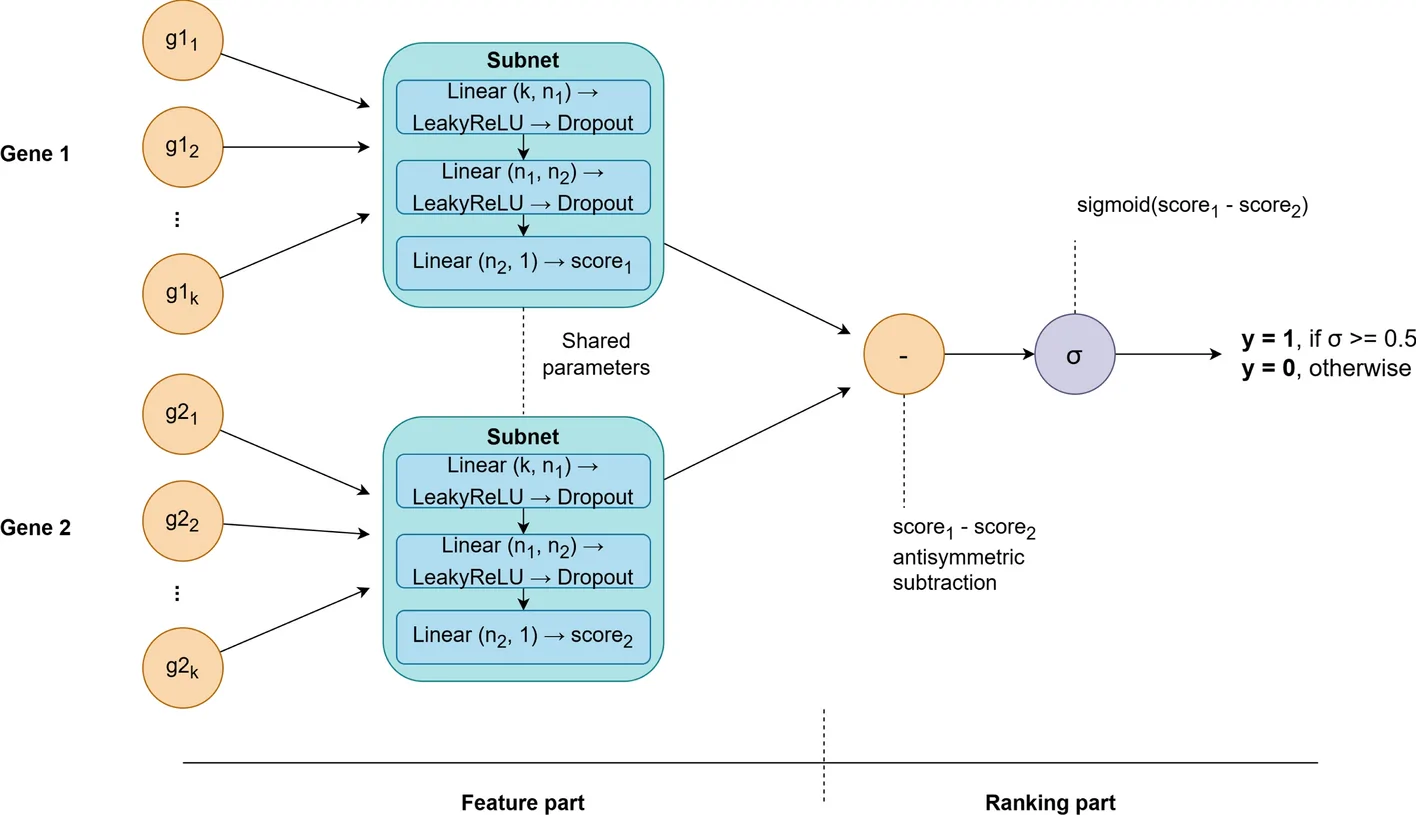
Pairwise ranking neural network architecture

We have developed a comparative model to assess the relative gene expression levels between two genes. Our model utilize a pairwise ranking approach: the histone marks of two genes are compared to determine which has a higher level of expression. The model was inspired by DirectRanker [8], and uses a neural network for pairwise ranking. The network architecture can be seen on Fig. 2. The full implementation can be found in our GitHub repository [21].

Our neural network consists of the following layers:

1. Input layer
  For the input generation, we divided the 10,000 base-pair (bp) DNA region (± 5,000 bp) around the TSS of each gene into bins of 100 bp. We then calculated the average peak value in each bin. For each histone mark, we obtained a single 100-bin matrix, and we constructed the input matrix from these.
  Because we performed an ablation study, the inputs were generated according to the current histone mark combination. Depending on which histone marks were included, we selected only those marks used by the model. For each gene, we combined the histone mark matrices into a single row of data by concatenating them. This made our input, *1 × 100* when using single histone mark, and *1 × 500* when using all five histone marks for each gene. After that, the histone mark matrices will be fed into neural network subnet.
2. Neural network subnet
  The DirectRanker was adopted for our pairwise ranking neural network architecture. This consist of two networks with the identical layer structures and shared parameters, processing Gene 1 and Gene 2 independently to produce $score_1$ and $score_2$ respectively. The difference between these two scores is then passed through a sigmoid function and discretised into a binary label *y*.
  - Hidden layer 1
    In the subnet, the first layer is hidden layer with size $k \times n_1$, where $n_1$ is the size of output of first hidden layer. This layer apply the linear transformation to the incoming data, mapping the input features to a new feature space.
    The linear transformation can be expressed as:
    1 $$\begin{aligned} y = x{A^T} + b \end{aligned}$$
    where *y* denotes the output of the first hidden layer, *x* i the weight matrix of $k \times n_1$, *A* represents the input featur vector, and *b* is the bias vector.
    We apply the non-linearity function called LeakyReLU. This will introduce nonlinearity in neural network and can help with vanishing gradient problem during training. It will enable the neural networks to learn more complex relationships in the data.
    The LeakyReLU can be expressed as:
    2 $$\begin{aligned} LeakyReLU(x)= & \max (0, x) + negative\_slope * \min (0, x) \text {, or} \end{aligned}$$
    3 $$\begin{aligned} LeakyReLU(x)= & {\left\{ \begin{array}{ll} x, & \text {if } x \ge 0 \\ negative\_slope \times x, & \text {otherwise} \end{array}\right. } \end{aligned}$$
    The next layer is dropout layer. This layer will randomly zeroes some of the elements of the input tensor with probability *p*. The dropout layer can avoid overfitting in the model, a common challenge in deep learning models. The dropout layer promotes the learning of more robust and generalized representations, and will enhance the model’s performance on unseen data.
  - Hidden layer 2
    Subsequent to the dropout layer, the data is processed through a second hidden layer. This layer is characterized by dimension $n_1 \times n_2$, where $n_1$ is the input size (output of the previous layer) and $n_2$ represents the number of output units in this second hidden layer. We also applied the LeakyReLU for this layer.
    To further enhance the model’s generalization capabilities, an additional dropout layer is incorporated following the second hidden layer. This layer employs the same dropout probability *p* as the first dropout layer, reinforcing the regularization effect throughout the network architecture.
  - Linear Layer
    The final layer of each subnet is a linear layer. This layer was characterized by dimension $n_2 \times 1$, where $n_2$ represents the number of output units from the second hidden layer. This layer produced a scalar score for each gene. This score is used in the subsequent asymmetric subtraction step.
3. Asymmetric Subtraction Layer
  The scalar scores from both subnets are subtracted to obtain a score difference. Due to the nature of twin-networks with shared parameters [8], this layer enforced the following condition for all $x, y, z \in \mathcal {F}$, where *r* is the score difference from neural network output.
  - Reflexivity
    4 $$\begin{aligned} r(x, x) = 0 \end{aligned}$$
  - Antisymmetry
    5 $$\begin{aligned} r(x, y) = -r(y, x) \end{aligned}$$
  - Transitivity
    6 $$\begin{aligned} (r(x, y) \ge 0 \wedge r(y, z) \ge 0) \Rightarrow r(x, z) \ge 0\nonumber \\ \end{aligned}$$
4. Output layer
  The final layer of the network architecture is a sigmoid function. The sigmoid function took the score difference from previous layer and used it to make class predictions by mapping it to a probability value in the range [0, 1], and can be expressed as:
  7 $$\begin{aligned} \sigma (x)=\frac{1}{1+e^{-x}} \end{aligned}$$
  This probability output then thresholded at 0.5 to make the final binary classification decision. The final result will be interpreted as follows:
  ‘1’, if the $$\sigma \ge 0.5$$,
  ‘0’, otherwise.

#### Baseline classifier

**Fig. 3:**
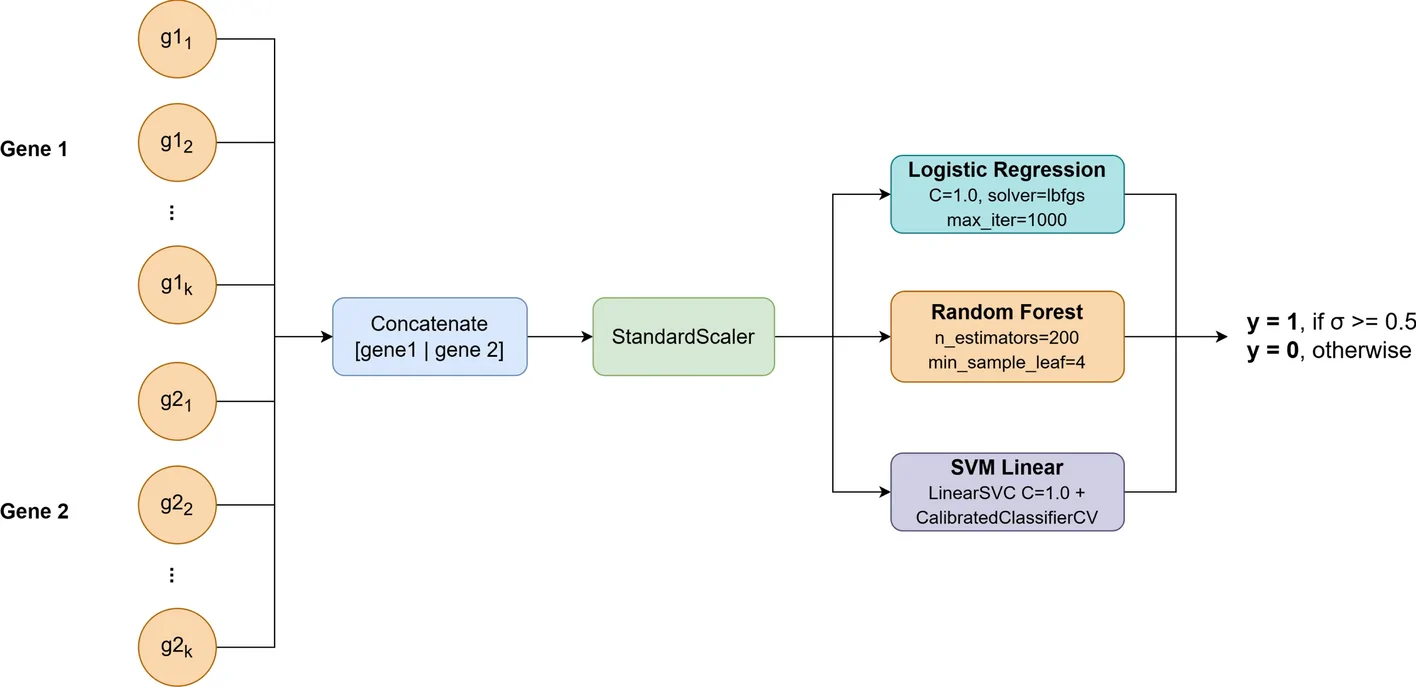
Baseline classifiers architecture

To evaluate whether the deep pairwise ranking network provides meaningful representation over simpler approaches, we implemented three baseline classifiers operating on identical input representations and data splits. The baseline classifier architecture can be seen on Fig. 3. Unlike the neural network, which processes each gene through independent subnets before performing antisymmetric subtraction, the baseline classifiers take a single concatenated feature vector as input.

1. Input Representation
  The feature vectors of Gene 1 and Gene 2 are concatenated into a single input vector [gene1 | gene2], following the same binning procedure. This yields an input of dimension *1 × 200* for a single histone mark and *1 × 1000* when using all five histone marks. The concatenated vector is then standardised using a StandardScaler, which normalises each feature to zero mean and unit variance prior to classification.
2. Classifier
  The same concatenated and scaled input is passed to each of the three baseline classifiers independently.
  - *Logistic Regression*: a linear classifier with regularisation strength C=1.0, trained using the L-BFGS solver with a maximum of 1,000 iterations.
  - *Random Forest*: an ensemble of 200 decision trees with a minimum of 4 samples per leaf, which reduces variance and prevents overfitting on small subsets.
  - *Support Vector Machine (Linear SVM)*: a LinearSVC with C=1.0, wrapped with CalibratedClassifierCV to produce probability outputs consistent with the other classifiers.
  All three classifiers produced a probability estimate that is thresholded at 0.5 to yield the final binary label *y=1* if Gene 1 has higher expression than Gene 2, and *y=0* otherwise.
  While the baseline classifiers share the same pairwise input construction and binary labelling scheme as the neural network, they differ in one fundamental aspect which is the concatenation-based approach does not enforce antisymmetry. Swapping the order of Gene 1 and Gene 2 in the input vector did not guaranteed to produce complementary predictions, meaning the classifiers may assign inconsistent rankings depending on input order. In contrast, the DirectRanker-inspired architecture explicitly enforces antisymmetry through its shared-parameter twin subnets and asymmetric subtraction layer, ensuring that *r(x, y) = -r(y, x)* by construction. The baseline classifiers are therefore included as reference points to assess the utility of the principled pairwise formulation rather than as pairwise rankers in their own right.

### Experiment setup

#### Histone marks choices

We investigated the impact of each type of histone mark during the classification and pairwise ranking processes. It is hypothesised that various histone mark types exert distinct influences on gene expression levels, either repressing or promoting transcription. In this study, five histone marks were used: H3K4me1, H3K4me3, H3K9ac, H3K27ac, and H3K27me3. To systematically assess the contribution of each mark, ablation testing was employed to evaluate all possible non-empty subsets of these five marks. Given five histone marks, this yields $2^5 - 1 = 31$ distinct combinations, ranging from individual marks tested in isolation to the full set of all five marks used together. This approach allows us to identify which histone marks, or combinations thereof, most significantly contribute to determining relative gene expression levels. The ablation test was conducted across all datasets.

#### Dataset partition

We partitioned the datasets into training (80%), validation (10%), and testing (10%) subsets. We implemented a strict non-leakage protocol wherein individual genes were confined to a single partition, thereby preventing any gene from appearing across multiple partitions and eliminating potential data contamination. Table 3 showed the number of genes in each dataset and their training, validation, and testing splits.

**Table 3:** Dataset splits used in the experiments

Dataset	Number of genes	Training set	Validation	Testing set
Donor 3	19,645	15,717	1964	1964
Donor 4	19,645	15,717	1964	1964
HepG2 GSE76344	22,154	17,724	2215	2215
HepG2 ENCSR134DWG	19,645	15,717	1964	1964

#### Gene pair generation and label distribution

During the training, gene pairs were generated separately for each partition (training, validation, and testing) using only the gene indices belonging to that partition, ensuring strict separation between splits. At each epoch, 8000 pairs were randomly sampled with replacement from the training partition, while 1000 pairs were sampled from each of the validation and testing partitions. For each sampled pair, the feature vector of Gene 1 and the feature vector of Gene 2 are fed independently into their respective subnets, producing $score_1$ and $score_2$. The binary label was assigned as *y = 1* if Gene 1 has a higher expression level than Gene 2, and *y = 0* otherwise. Sampling with replacement was used to maximise the combinatorial diversity of pairs at each epoch, given that the number of possible unique pairs far exceeds the number of genes in each partition.

Since gene pairs are constructed by randomly sampling two genes from the same partition, the resulting label distribution is approximately balanced by design. For any randomly drawn pair, the probability of Gene 1 having higher expression than Gene 2 is roughly equal to the probability of the reverse, yielded a near 50:50 split between *y = 1* and *y = 0* across the sampled pairs. To confirm this, Table 4 reported the exact label distribution across training, validation, and test pairs for each dataset, showing the number and proportion of positive (*y = 1*) and negative (*y = 0*) pairs. This balanced distribution means that accuracy is a meaningful metric and that the model is not biased towards a majority class.

**Table 4:** Class distribution across dataset splits

Dataset	Training set		Validation set		Test set	
	Class 0	Class 1	Class 0	Class 1	Class 0	Class 1
Donor 3	50.99%	49.01%	52.04%	47.96%	50.72%	49.28%
Donor 4	50.99%	49.01%	52.04%	47.96%	50.72%	49.28%
HepG2 GSE76344	52.72%	47.28%	52.32%	47.68%	51.44%	48.56%
HepG2 ENCSR134DWG	51.59%	48.41%	53.3%	46.7%	50.98%	49.02%

#### Training objectives

The model is trained by minimizing a composite loss function consisting of three terms: binary cross-entropy, regularization penalties, and an antisymmetry auxiliary loss. The binary cross-entropy loss is computed as the mean over all samples in a batch:

8 $$\begin{aligned} L_{\text {BCE}} = -\frac{1}{N} \sum _{i} \left[ y_i \log (\sigma _i) + (1 - y_i) \log (1 - \sigma _i) \right] \end{aligned}$$

where *N* is the batch size, $y_i \in \{0, 1\}$ is the ground-truth binary label for the *i*-th pair indicating whether Gene 1 has higher expression than Gene 2, and $\sigma _1$ is the corresponding sigmoid output of the network.

To discourage overly large weights and improve generalisation, *L*1 and *L*2 regularisation penalties are applied to all weight matrices in the network:

9 $$\begin{aligned} L_{reg} = \lambda _1 \cdot \sum \Vert w\Vert _1 + \lambda _2 \cdot \sum \Vert w\Vert _2 \end{aligned}$$

where $\lambda _1 = 0.005$ and $\lambda _2 = 0.001$ are the *L*1 and *L*2 regularisation coefficients respectively, $\Vert w\Vert _1$ denotes the *L*1 norm (sum of absolute values), and $\Vert w\Vert _2$ denotes the *L*2 spectral norm (largest singular value) of each weight matrix.

To further reinforce antisymmetric behaviour during training, an auxiliary antisymmetry loss is added. For each pair (Gene 1, Gene 2), the swapped pair (Gene 2, Gene 1) is also passed through the network, and the model is penalised if the two predictions do not sum to 1:

10 $$\begin{aligned} L_{antisym} = \text {mean}\left( \sigma (x, y) + \sigma (y, x) -1 \right) ^2 \end{aligned}$$

Then the final total loss is:

11 $$\begin{aligned} L_{total} = L_{BCE} + L_{reg} + \lambda _{antisym}\cdot L_{antisym} \end{aligned}$$

where $\lambda _{antisym} = 0.5$ is the antisymmetry loss weight.

#### Training parameters

The model was optimised using Stochastic Gradient Descent (SGD) with a momentum of 0.9 and an initial learning rate of 0.0001. The network consists of two hidden layers with 64 and 32 units respectively, a dropout rate of 0.3, and a LeakyReLU negative slope of 0.1. Training was conducted for a maximum of 100 epochs with a batch size of 32. At each epoch, 8000 pairs were drawn for training, 1000 for validation, and 1000 for testing, all sampled with replacement from their respective gene-level partitions.

To prevent overfitting and ensure efficient convergence, two adaptive training strategies were employed. First, a ReduceLROnPlateau scheduler monitored the validation loss and reduced the learning rate by a factor of 0.1 if no improvement was observed for 5 consecutive epochs. Second, early stopping was implemented with a patience of 10 epochs and a minimum improvement threshold of $1 \times 10^{-4}$, halting training if the validation loss failed to improve and restoring the model weights to those of the best-performing epoch. All experiments were repeated across 5 independent random seeds to assess stability, and results are reported as the mean and standard deviation across these runs.

## Results

### Evaluation metrics

To assess model performance, three evaluation metrics were used: classification accuracy, the Area Under the Receiver Operating Characteristic Curve (AUROC), and the Area Under the Precision–Recall Curve (AUPRC).

Accuracy measures the proportion of correctly classified gene pairs out of all evaluated pairs:

12 $$\begin{aligned} \text {Accuracy} = \frac{TP + TN}{TP + TN + FP + FN} \end{aligned}$$

where *TP* (true positive) and *TN* (true negatives) are correctly pairs, and *FP* (false positives) and *FN* (false negatives are misclassified pairs.

AUROC summarises the trade-off between the true positive rate (*TPR*, also called sensitivity or recall) and the false positive rate (*FPR*) across all classification thresholds. *TPR* and *FPR* are defined as:

13 $$\begin{aligned} \text {TPR} = \frac{TP}{TP + FN}, \qquad \text {FPR} = \frac{FP}{FP + TN} \end{aligned}$$

The AUROC is then computed as the area under the ROC curve:

14 $$\begin{aligned} \text {AUROC} = \int _0^1 \text {TPR}(\text {FPR}) \, d(\text {FPR}) \end{aligned}$$

A value of 1.0 indicates perfect discrimination, while 0.5 corresponds to a random classifier. Unlike accuracy, AUROC is threshold-independent and robust to class imbalance.

AUPRC summarises the trade-off between precision and recall across all thresholds. Precision and recall are defined as:

15 $$\begin{aligned} \text {Precision} = \frac{TP}{TP + FP}, \qquad \text {Recall} = \frac{TP}{TP + FN} \end{aligned}$$

The AUPRC is computed as the area under the precision–recall curve:

16 $$\begin{aligned} \text {AUPRC} = \int _0^1 \text {Precision}(\text {Recall}) \, d(\text {Recall}) \end{aligned}$$

AUPRC is particularly informative for imbalanced datasets, as it focuses on model performance on the positive class. A random classifier achieves an AUPRC equal to the proportion of positive samples in the dataset.

### Training dynamics

**Fig. 4:**
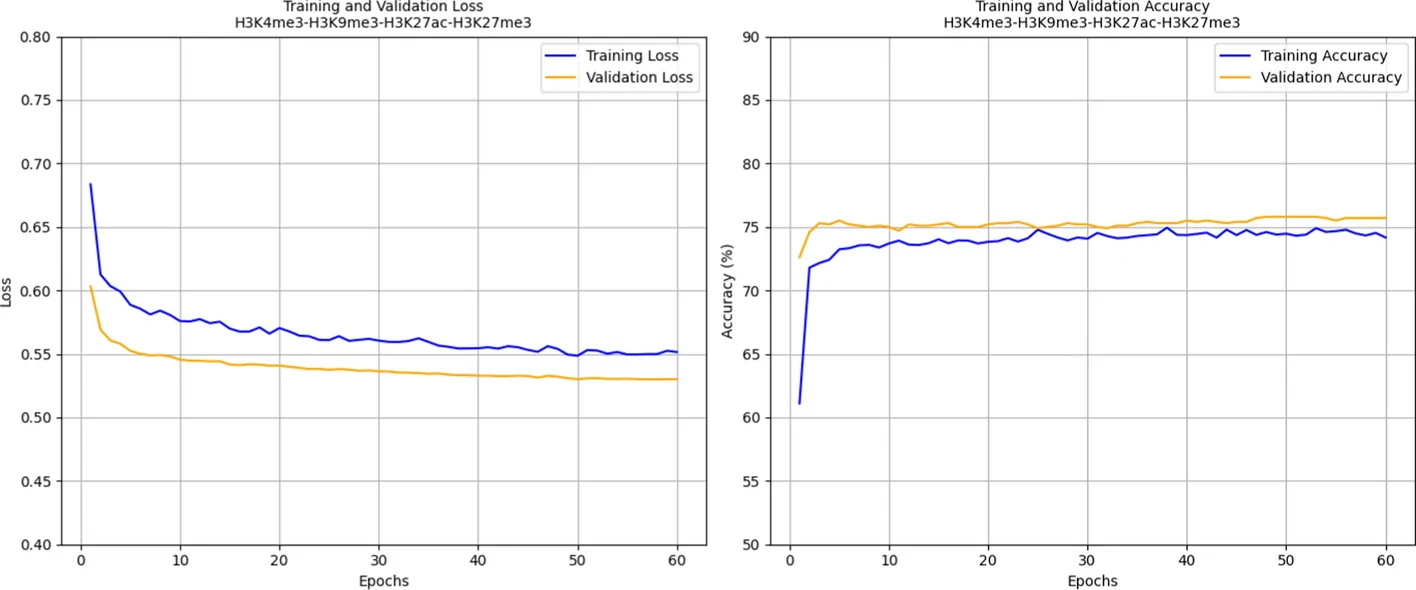
Training and validation loss and accuracy of Donor 3-H3K4me3-H3K9me3-H3K27ac-H3K27me3 (seed 42)

**Fig. 5:**
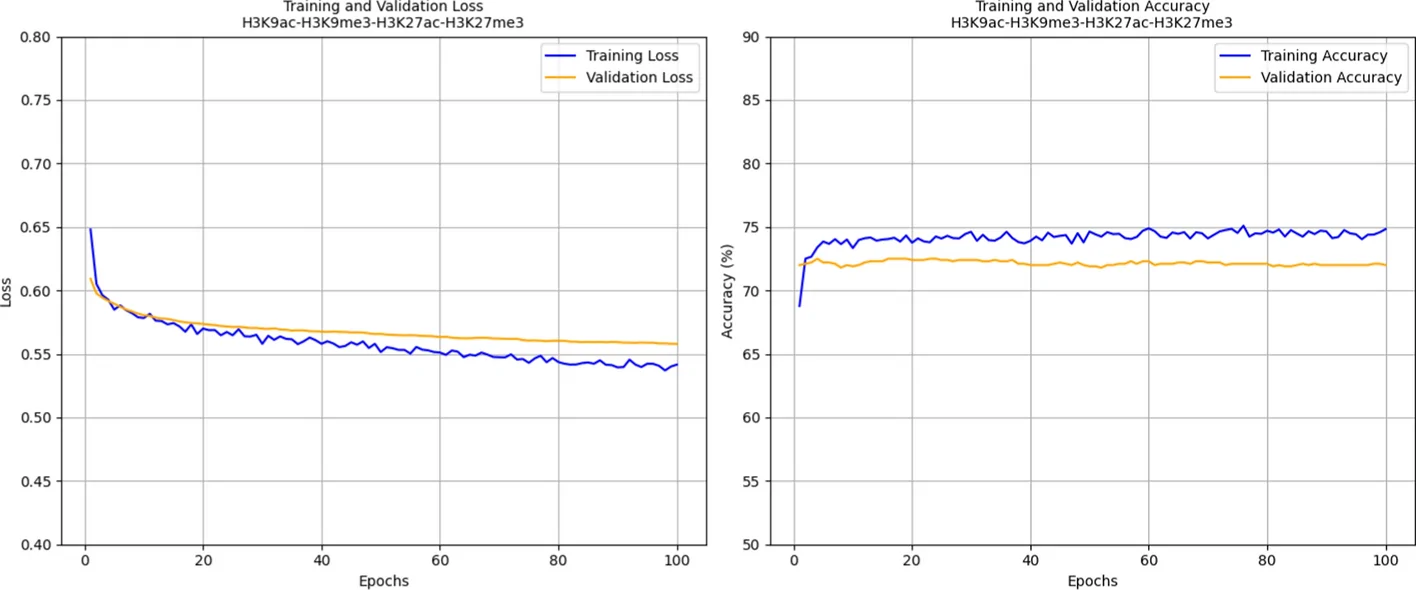
Training and validation loss and accuracy of Donor 4-H3K9ac-H3K9me3-H3K27ac-H3K27me3 (seed 789)

**Fig. 6:**
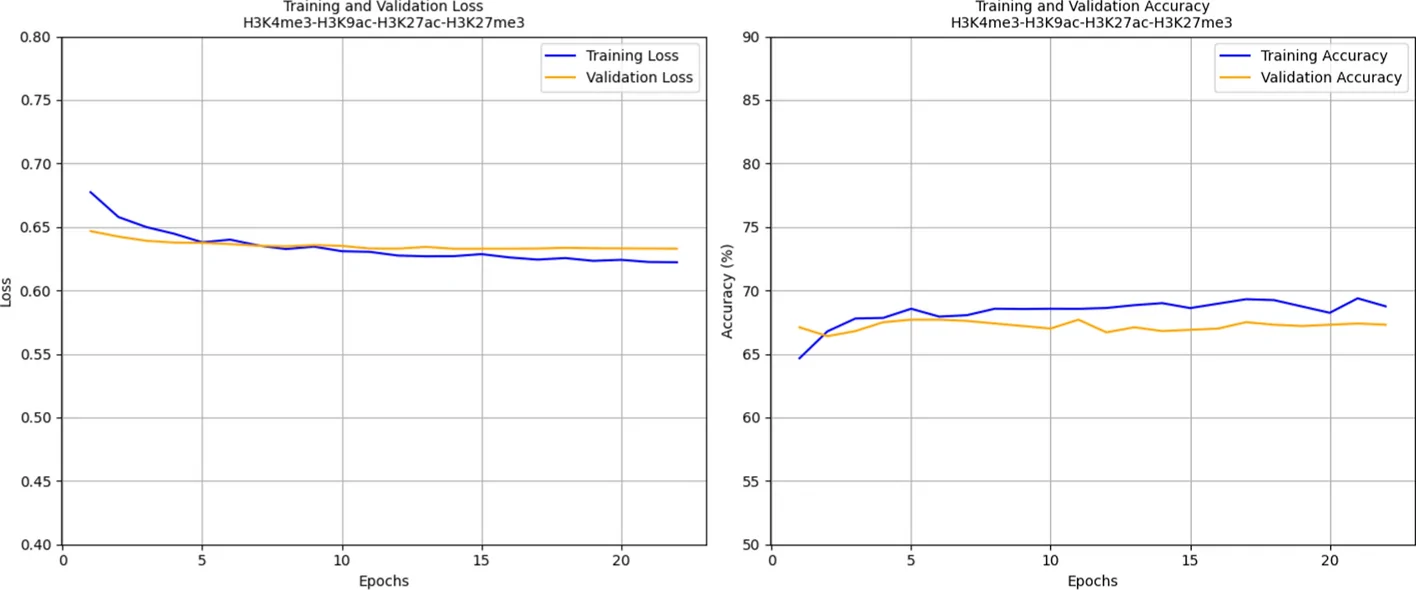
Training and validation loss and accuracy of HepG2 GSE76344-H3K4me3-H3K9ac-H3K27ac-H3K27me3 (seed 123)

**Fig. 7:**
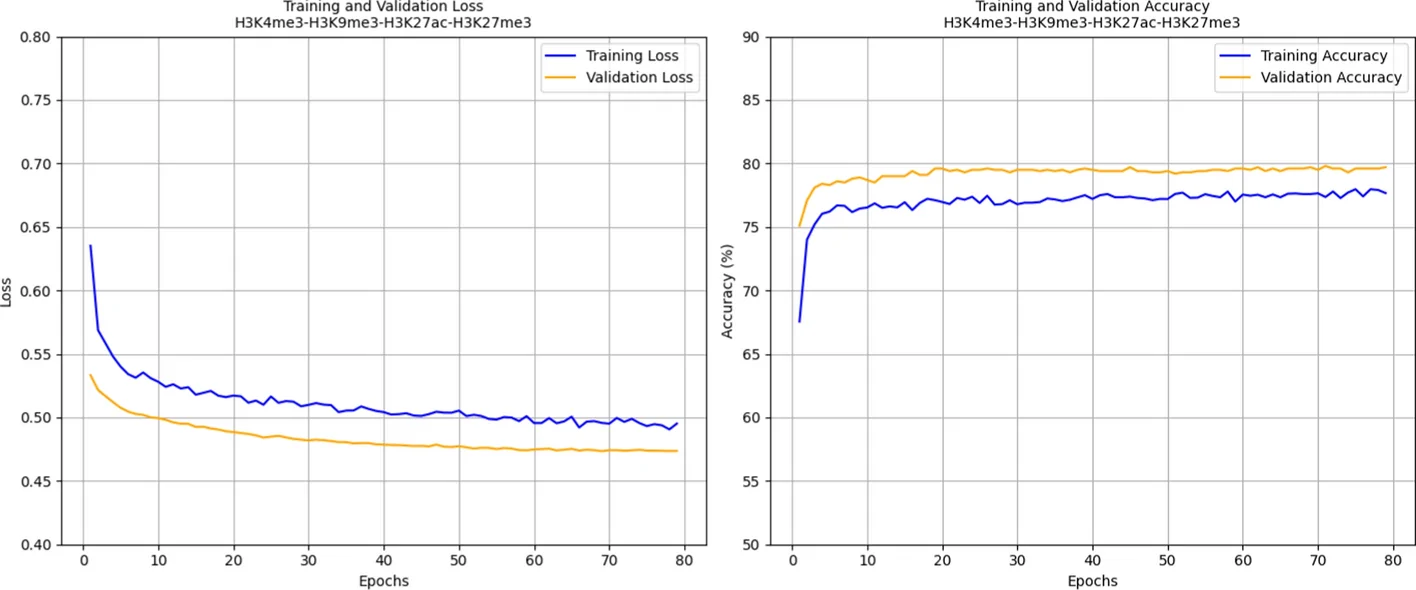
Training and validation loss and accuracy of HepG2 ENCSR134DWG-H3K4me3-H3K9me3-H3K27ac-H3K27me3 (seed 456)

The pairwise ranking network demonstrated stable and consistent convergence across all four datasets. Figure 4, 5, 6 and Fig. 7 showed representative training and validation loss and accuracy curves for each dataset (one seed shown per dataset). In all cases, the training loss decreased rapidly within the first 10–20 epochs before stabilising, while validation loss closely tracked training loss with no substantial divergence, indicating that the early stopping and ReduceLROnPlateau strategies effectively prevented overfitting. Validation accuracy consistently converged to values in the 65–80% range depending on the histone mark combination and dataset, and the training and validation curves remained tightly coupled throughout, reflecting good generalisation.

The number of epochs before convergence varied across datasets. Donor 3 converged in approximately 60 epochs, Donor 4 in approximately 100 epochs, HepG2 GSE76344 in approximately 20 epochs, and HepG2 ENCSR134DWG in approximately 80 epochs. The notably faster convergence for HepG2 GSE76344 is consistent with its relatively lower predictive performance (discussed below), suggesting a shallower loss landscape for that dataset.

### Discriminative performance: AUROC and AUPRC

**Fig. 8:**
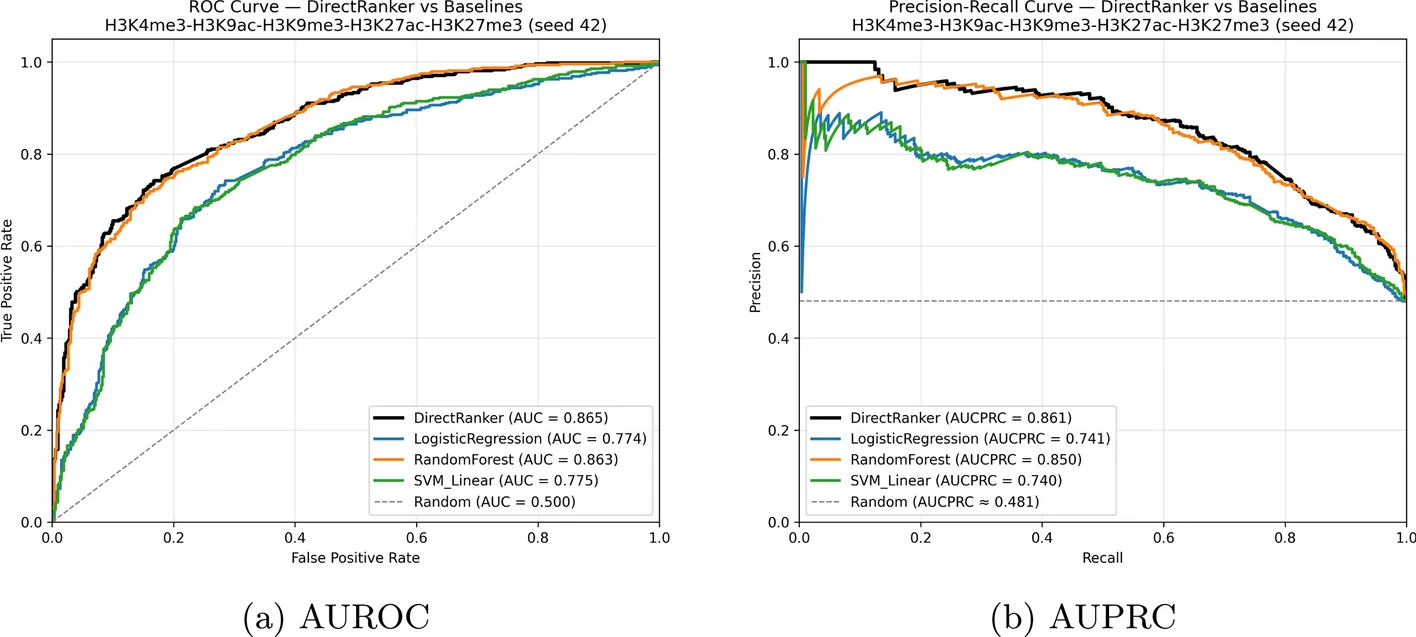
Receiver operating characteristic curve (AUROC) and precision-recall curve (AUPRC) for Donor 3: H3K4me3, H3K9ac, H3K9me3, H3K27ac, H3K27me3 (seed 42)

**Fig. 9:**
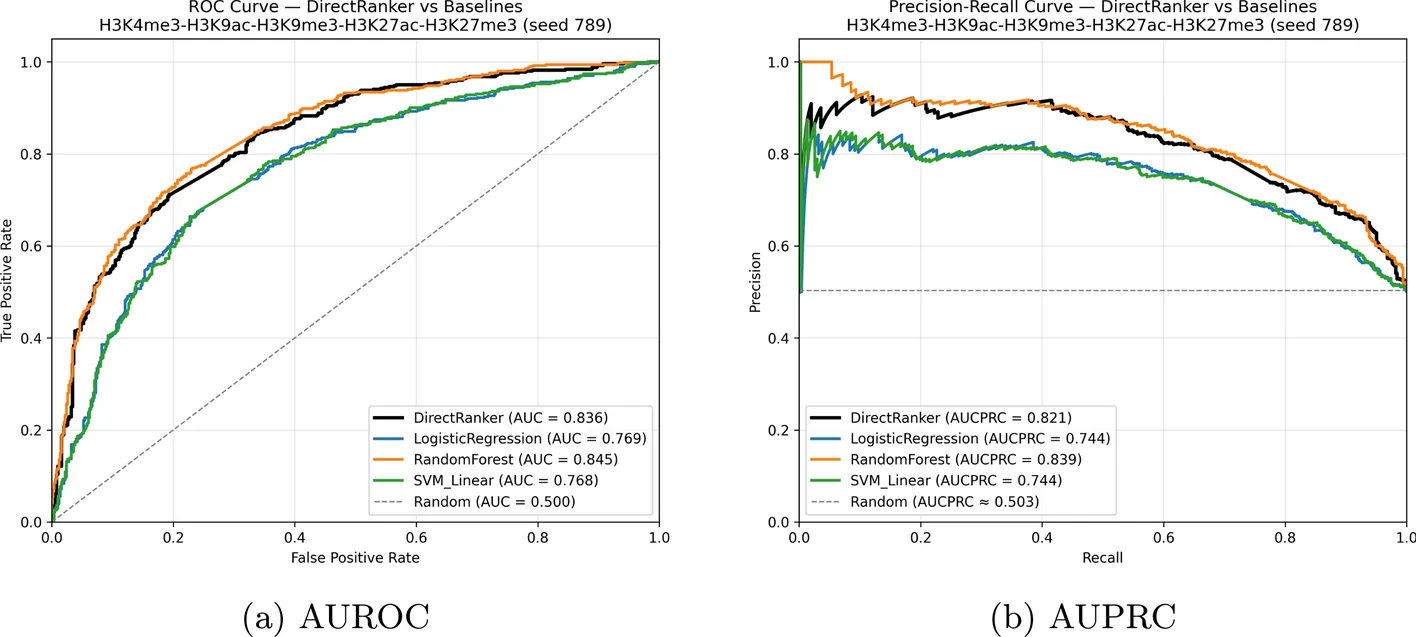
Receiver operating characteristic curve (AUROC) and precision-recall curve (AUPRC) for Donor 4: H3K4me3, H3K9ac, H3K9me3, H3K27ac, H3K27me3 (seed 789)

**Fig. 10:**
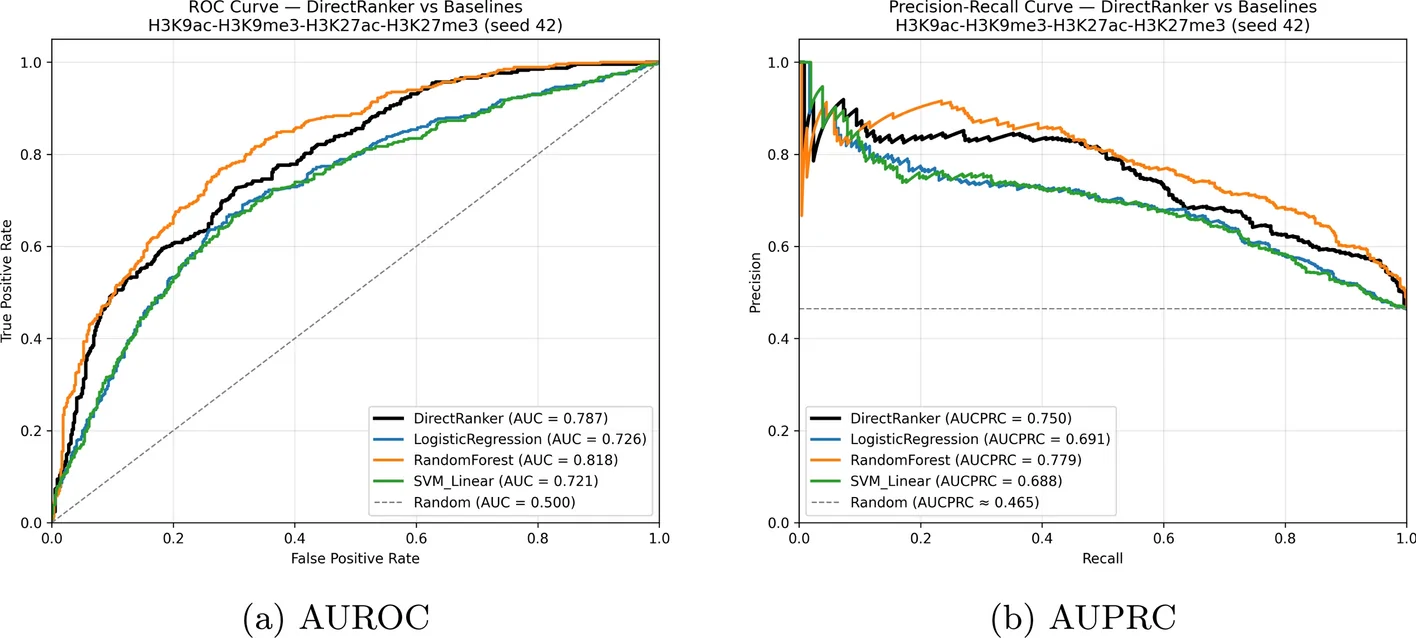
Receiver operating characteristic curve (AUROC) and precision-recall curve (AUPRC) for HepG2 (GSE76344): H3K9ac, H3K9me3, H3K27ac, H3K27me3 (seed 42)

**Fig. 11:**
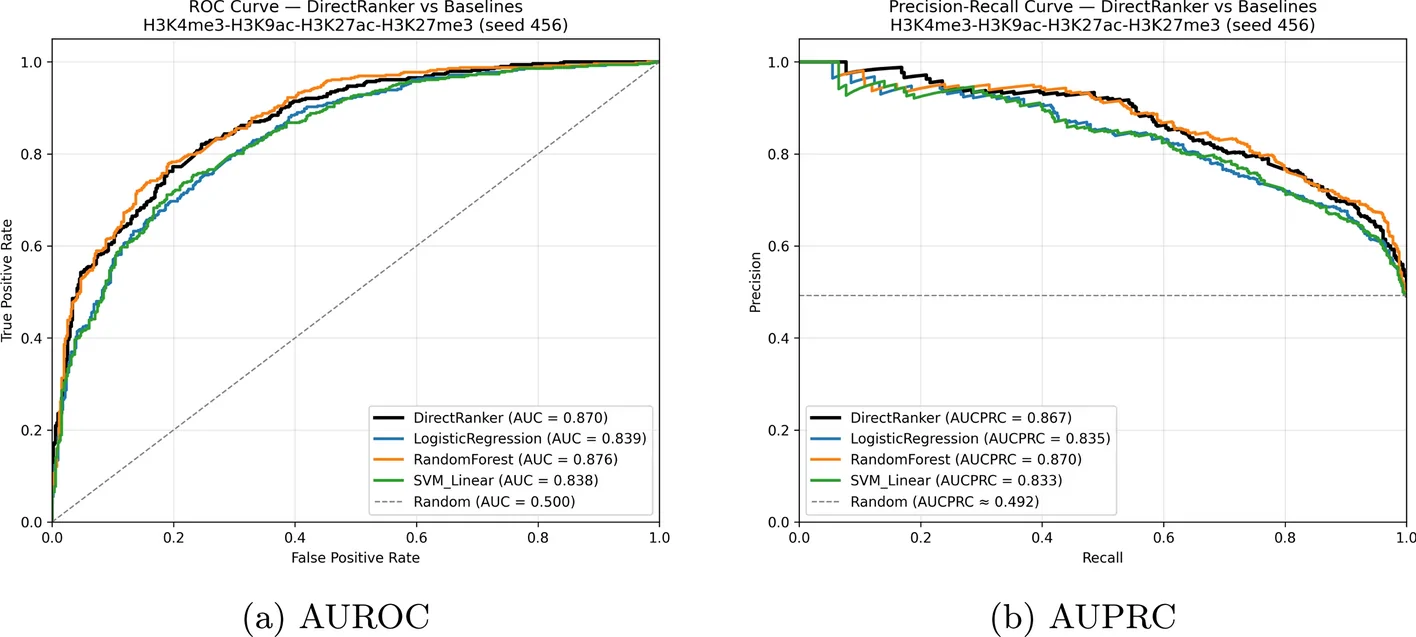
Receiver Operating Characteristic Curve (AUROC) and precision-recall curve (AUPRC) for HepG2 (ENCSR134DWG): H3K4me3, H3K9ac, H3K27ac, H3K27me3 (seed 456)

Figures 8, 9, 10 and 11 showed the Receiver Operating Characteristic (ROC) and Precision–Recall curves for DirectRanker against the three baseline classifiers on all four datasets, evaluated on the best-performing histone mark combination per dataset (all five marks combined or the best ablation subset). A random classifier baseline (AUROC = 0.500) is included in each panel for reference.

**Donor 3**. For the five-mark combination (H3K4me3, H3K9ac, H3K9me3, H3K27ac, H3K27me3) at seed 42, DirectRanker achieved an AUROC of 0.865 and an AUPRC of 0.861, closely matching Random Forest (AUROC = 0.863, AUPRC = 0.850) while outperforming Logistic Regression (AUROC = 0.774, AUPRC = 0.741) and SVM Linear (AUROC = 0.775, AUPRC = 0.740). Across five independent seeds, the mean test AUROC for DirectRanker was 0.839 ± 0.016 and AUPRC was 0.834 ± 0.017 (Table 5).

**Donor 4**. At seed 789, DirectRanker achieved an AUROC of 0.836 and AUPRC of 0.821 for the five-mark combination, compared to Random Forest (AUROC = 0.845, AUPRC = 0.839), Logistic Regression (AUROC = 0.769, AUPRC = 0.744), and SVM Linear (AUROC = 0.768, AUPRC = 0.744). Averaged over five seeds, DirectRanker attained a mean test AUROC of 0.833 ± 0.007 and AUPRC of 0.819 ± 0.010 on its best combination (H3K4me3-H3K9ac-H3K27ac-H3K27me3) (Table 6).

**HepG2 GSE76344**. This dataset was more challenging for all models. At seed 42, DirectRanker with H3K9ac-H3K9me3-H3K27ac-H3K27me3 achieved an AUROC of 0.787 and AUPRC of 0.750, compared to Random Forest (AUROC = 0.818, AUPRC = 0.779), Logistic Regression (AUROC = 0.726, AUPRC = 0.691), and SVM Linear (AUROC = 0.711, AUPRC = 0.688) (Fig. 10a). Across five seeds, DirectRanker’s best combination (H3K4me3-H3K9me3-H3K27ac-H3K27me3) yielded a mean test AUROC of 0.777 ± 0.023 and AUPRC of 0.739 ± 0.021 (Table 7). The lower performance of all models on this dataset relative to ENCSR134DWG likely reflects differences in sequencing depth and library preparation between the two HepG2 sources.

**HepG2 ENCSR134DWG**. This dataset yielded the strongest overall results. At seed 456, DirectRanker with H3K4me3-H3K9ac-H3K27ac-H3K27me3 achieved an AUROC of 0.870 and AUPRC of 0.967, outperforming the single-seed ROC values of Random Forest (AUROC = 0.876, AUPRC = 0.870), Logistic Regression (AUROC = 0.839, AUPRC = 0.835), and SVM Linear (AUROC = 0.838, AUPRC = 0.833) (Fig. 11). Across five seeds, the mean test AUROC for DirectRanker was 0.856 ± 0.018 and AUPRC was 0.856 ± 0.021 (Table 8), the highest AUPRC among all models tested on this dataset.

### Classification performance

Tables 5, 6, 7 and 8 summarise the best-performing histone mark combination per model and per dataset, averaged across five independent seeds (full results for all 31 combinations are available in Additional files 3–6). For the two normal liver cell datasets (Donors 3 and 4), Random Forest consistently achieved the highest test accuracy ( 76.16–76.54%) and AUROC ( 0.844–0.845). DirectRanker attained test accuracies of 74.30–74.56% and AUROCs of 0.833–0.839, performing below Random Forest by approximately 1.5–2 percentage points in accuracy but within comparable range in terms of discriminative ability. The two linear baselines (Logistic Regression and SVM Linear) achieved lower test accuracies (73.9–74.0%) and AUROCs ( 0.808–0.810), confirming that the nonlinear models provide a meaningful advantage.

For HepG2 ENCSR134DWG, the performance gap between DirectRanker and Random Forest narrowed considerably. Random Forest achieved the highest test accuracy (77.84 ± 1.15%) and AUROC (0.867 ± 0.013), while DirectRanker reached 76.38 ± 1.40% accuracy and 0.856 ± 0.018 AUROC—a difference of approximately 1.5 percentage points—while achieving the highest AUPRC among all models (0.856 ± 0.021 vs. Random Forest’s 0.861 ± 0.008). For HepG2 GSE76344, Random Forest retained the highest accuracy (71.90 ± 1.54%) and AUROC (0.797 ± 0.017), with DirectRanker’s best combination achieving 69.64 ± 1.63% accuracy and AUROC 0.777 ± 0.023.

**Table 5:** Performance of the best-performing histone mark combination per model on the **Donor 3** dataset

Model	Best histone combination	Accuracy (%)	AUROC	AUPRC	Antisymmetry
DirectRanker	H3K4me3-H3K9ac-H3K9me3-H3K27ac-H3K27me3	74.56 ± 1.63	0.839 ± 0.016	0.834 ± 0.017	**0.961 ± 0.003**
Random Forest	H3K4me3-H3K9ac-H3K9me3-H3K27ac-H3K27me3	**76.16 ± 1.43**	**0.845 ± 0.015**	**0.832 ± 0.015**	0.851 ± 0.009
Logistic Regression	H3K27ac	74.30 ± 1.44	0.809 ± 0.015	0.793 ± 0.016	0.790 ± 0.021
SVM Linear	H3K27ac	73.941.55	0.810 ± 0.016	0.793 ± 0.015	0.787 ± 0.019

Results are mean ± SD across five independent random seeds. Best value per column in bold

**Table 6:** Performance of the best-performing histone mark combination per model on the **Donor 4** dataset

Model	Best histone combination	Accuracy (%)	AUROC	AUPRC	Antisymmetry
DirectRanker	H3K4me3-H3K9ac-H3K27ac-H3K27me3	74.30 ± 0.69	0.833 ± 0.007	0.819 ± 0.010	**0.918 ± 0.008**
Random Forest	H3K4me3-H3K9ac-H3K9me3-H3K27ac-H3K27me3	**76.54 ± 1.01**	**0.844 ± 0.008**	**0.830 ± 0.015**	0.841 ± 0.010
Logistic Regression	H3K4me3-H3K27ac	73.98 ± 1.37	0.808 ± 0.013	0.793 ± 0.015	0.776 ± 0.014
SVM Linear	H3K4me3-H3K27ac	73.60 ± 1.31	0.809 ± 0.014	0.794 ± 0.014	0.775 ± 0.012

Results are mean ± SD across five independent random seeds. Best value per column in bold

**Table 7:** Performance of the best-performing histone mark combination per model on the **HepG2 GSE76344** dataset

Model	Best histone combination	Accuracy (%)	AUROC	AUPRC	Antisymmetry
DirectRanker	H3K4me3-H3K9me3-H3K27ac-H3K27me3	69.64 ± 1.63	0.777 ± 0.023	0.739 ± 0.021	**0.983 ± 0.006**
Random Forest	H3K9ac-H3K9me3-H3K27ac-H3K27me3	**71.90 ± 1.54**	**0.797 ± 0.017**	**0.769 ± 0.019**	0.772 ± 0.011
Logistic Regression	H3K4me3	69.34 ± 1.69	0.744 ± 0.021	0.685 ± 0.028	0.750 ± 0.018
SVM Linear	H3K4me3	69.42 ± 1.70	0.745 ± 0.021	0.686 ± 0.028	0.745 ± 0.014

Results are mean ± SD across five independent random seeds. Best value per column in bold

**Table 8:** Performance of the best-performing histone mark combination per model on the **HepG2 ENCSR134DWG** dataset

Model	Best histone combination	Accuracy (%)	AUROC	AUPRC	Antisymmetry
DirectRanker	H3K4me3-H3K9ac-H3K27ac-H3K27me3	76.38 ± 1.40	0.856 ± 0.018	0.856 ± 0.021	**0.978 ± 0.004**
Random Forest	H3K4me3-H3K9ac-H3K27ac-H3K27me3	**77.84 ± 1.15**	**0.867 ± 0.013**	**0.861 ± 0.008**	0.842 ± 0.021
Logistic Regression	H3K9ac	76.04 ± 2.04	0.834 ± 0.020	0.820 ± 0.022	0.763 ± 0.009
SVM Linear	H3K4me3-H3K9ac	75.82 ± 1.16	0.835 ± 0.018	0.821 ± 0.025	0.768 ± 0.006

Results are mean *v* SD across five independent random seeds. Best value per column in bold

### Antisymmetry compliance

A key theoretical property of the DirectRanker architecture is its enforcement of antisymmetry, that is, the prediction for gene pair (A, B) should be the complement of the prediction for pair (B, A). This property is structurally guaranteed by the asymmetric subtraction layer in the network, which by construction satisfies *r(x, y) = -r(y, x)*. The antisymmetry metric reported in Tables 5, 6, 7 and 8 quantifies how consistently this property holds in practice; full per-combination antisymmetry scores are reported in Additional files 3–6.

DirectRanker achieved substantially higher antisymmetry scores than all three baselines across every dataset. On Donor 3, the mean antisymmetry for DirectRanker was 0.961 ± 0.003 (best combination), compared to 0.851 ± 0.009 for Random Forest, 0.790 ± 0.021 for Logistic Regression, and 0.787 ± 0.019 for SVM Linear. Similar patterns were observed across Donor 4 (DirectRanker: 0.918 ± 0.008), HepG2 ENCSR134DWG (DirectRanker: 0.978 ± 0.004), and HepG2 GSE76344 (DirectRanker: 0.983 ± 0.006). This confirms that the pairwise ranking formulation not only matches or approaches the discriminative performance of Random Forest, but also produces more logically consistent, antisymmetric predictions which critical requirement for ranking applications.

### Impact of histone mark combinations

The ablation study across all 31 non-empty subsets of the five histone marks revealed consistent patterns across datasets (see Additional file 2). Combinations that included H3K27ac, a well-established mark of active enhancers and promoters, consistently ranked among the top performers. Promoter-associated marks (H3K4me3 and H3K9ac) further boosted performance when added to H3K27ac, and the full five-mark combination generally performed at or near the top for DirectRanker and Random Forest.

In contrast, marks associated with repressive chromatin (H3K9me3 and H3K27me3) consistently underperformed when used in isolation. Across all four datasets, single-mark models for H3K9me3 achieved test accuracies of 50–52% and AUROC values of 0.50–0.53, while H3K27me3 fared somewhat better at 54–66% accuracy and AUROC 0.55–0.72. Both repressive marks thus fell within the 50–60% accuracy range across models and datasets, consistently below all active mark combinations. These results suggest that repressive marks, while functionally important in silencing gene expression, do not independently encode sufficient information to rank relative expression levels, and their contribution to model performance is primarily realised through their combinatorial relationship with active marks.

The top-performing combinations for DirectRanker across datasets were enriched for promoter and active marks: H3K4me3-H3K9ac-H3K9me3-H3K27ac-H3K27me3 and H3K4me3-H3K9ac-H3K27ac-H3K27me3 consistently ranked in the top five across all four datasets, suggesting that a four-mark combination excluding H3K9me3 may offer comparable performance with reduced feature dimensionality.

## Discussion

The results confirm that promoter-proximal histone modification signals carry sufficient information to rank relative gene expression levels without relying on arbitrary expression thresholds. Across all four liver-derived datasets, multi-mark combinations anchored by active marks (H3K27ac, H3K4me3, H3K9ac) consistently drove predictive performance, consistent with their established roles at actively transcribed promoters. Repressive marks (H3K9me3, H3K27me3) contributed limited discriminative power in isolation, reflecting that broad heterochromatin domains have a less direct relationship to individual gene expression levels. The observed performance plateau when adding more active marks is better interpreted as correlated predictive signal within the promoter-proximal feature space, rather than biological redundancy among the marks themselves.

While Random Forest achieved marginally higher accuracy than DirectRanker on most datasets, the key advantage of the pairwise ranking formulation lies in its structural antisymmetry guarantee. Baseline classifiers do not inherently ensure that swapping a gene pair produces complementary predictions, a property they only partially achieve in practice. DirectRanker enforces this by construction, making it the more principled choice for applications that require a globally coherent gene ranking rather than isolated pairwise classifications.

The performance gap between the two HepG2 datasets, despite originating from the same cell line, likely reflects technical differences in ChIP-seq library preparation and sequencing depth between the ENCODE-standardised ENCSR134DWG and the independently generated GSE76344. This highlights the sensitivity of epigenomics models to data quality and the importance of standardised protocols when benchmarking across studies.

Several limitations should be noted. The model captures only promoter-proximal signals within a ±5,000 bp window and therefore does not account for distal enhancers, which can regulate expression over distances exceeding 1 Mb. Gene-level partitioning also does not account for topologically associating domains (TADs), where co-regulated genes may share epigenetic environments across training and test splits. Finally, all four datasets are liver-derived, and cross-tissue generalisation of the framework remains unevaluated. Future work incorporating long-range regulatory elements, chromosome-level data splits, and broader tissue representation would address these limitations and establish the broader utility of pairwise ranking for epigenomics.

## Conclusions

This study presented a deep learning framework based on pairwise ranking to predict relative gene expression levels from histone modification signals, evaluated across two normal liver and two hepatocellular carcinoma datasets. The proposed DirectRanker architecture achieved AUROC values of 0.833−0.867 and test accuracies of 74-78% across datasets, while structurally enforcing antisymmetry in its predictions, a property that baseline classifiers only partially satisfy and which is essential for producing logically consistent gene rankings. Active promoter-associated marks (H3K27ac, H3K4me3, and H3K9ac) were the primary drivers of predictive performance, while repressive marks (H3K9me3 and H3K27me3) contributed limited discriminative power in isolation, with single-mark models achieving test accuracies of only 50-66%. The observed performance saturation with increasing numbers of active marks reflects correlated predictive signal within the promoter-proximal feature space, rather than biological redundancy among the marks themselves.

The pairwise ranking formulation offers a principled alternative to threshold-based classification by enabling relative expression comparisons without the need for arbitrary binarisation cutoffs. While the current study is limited to liver-derived datasets and promoter-proximal signals, the framework is readily extensible to other tissue types and could be enhanced through the incorporation of distal regulatory elements. These directions represent natural next steps toward a more comprehensive model of epigenetic regulation of gene expression.

## Additional file

Additional file 1.

Additional file 2.

Additional file 3.

Additional file 4.

Additional file 5.

Additional file 6.

## Data Availability

The datasets are publicly available at NCBI GEO (GSE76344 and GSE19465) and ENCODE (ENCSR134DWG) as mentioned in the dataset section in Table 1 [16-19]. The source code is available at GitHub [22].

## References

[1] Weinhold B. Epigenetics: The science of change. Environ Health Perspect. 2006;114:160. 10.1289/EHP.114-A160. PMC1392256 16507447

[2] Mattei AL, Bailly N, Meissner A. Dna methylation: a historical perspective. Trends Genet. 2022;38:676–707. 10.1016/J.TIG.2022.03.010. 35504755

[3] Tompkins JD. Discovering dna methylation, the history and future of the writing on dna. J Hist Biol. 2022;55:865–87. 10.1007/S10739-022-09691-8/FIGURES/1. PMC9941238 36239862

[4] Singh R, Lanchantin J, Robins G, Qi Y. Deepchrome: deep-learning for predicting gene expression from histone modifications. Bioinformatics. 2016;32:639–48. 10.1093/BIOINFORMATICS/BTW427. 27587684

[5] Kadavath S, Paradis S, Yeung J. Deepchrome 2.0: Investigating and improving architectures, visualizations, & experiments (2022). 10.48550/arXiv.2209.11923

[6] Burges C, Shaked T, Renshaw E, Lazier A, Deeds M, Hamilton N, Hullender G. Learning to rank using gradient descent. In: Proceedings of the 22nd International Conference on Machine Learning. ICML ’05, pp. 89–96. Association for Computing Machinery, New York, NY, USA (2005). 10.1145/1102351.1102363.

[7] Burges CJC. From ranknet to lambdarank to lambdamart: An overview. Technical Report MSR-TR-2010-82 (June 2010). https://www.microsoft.com/en-us/research/publication/from-ranknet-to-lambdarank-to-lambdamart-an-overview/

[8] Köppel M, Segner A, Wagener M, Pensel L, Karwath A, Kramer S. Pairwise learning to rank by neural networks revisited: Reconstruction, theoretical analysis and practical performance. In: Brefeld U, Fromont E, Hotho A, Knobbe A, Maathuis M, Robardet C, editors. Machine Learning and Knowledge Discovery in Databases. Cham: Springer; 2020. p. 237–52.

[9] Frasca F, Matteucci M, Leone M, Morelli MJ, Masseroli M. Accurate and highly interpretable prediction of gene expression from histone modifications. BMC Bioinform. 2022;23:151. 10.1186/S12859-022-04687-X. PMC9040271 35473556

[10] Singh R, Lanchantin J, Sekhon A, Qi Y. Attend and predict: Understanding gene regulation by selective attention on chromatin. Adv Neural Inf Process Syst. 2017;30:6785. PMC6105294 30147283

[11] Chen Y, Xie M, Wen J. Predicting gene expression from histone modifications with self-attention based neural networks and transfer learning. Front Genet. 2022;13:1081842. 10.3389/FGENE.2022.1081842/BIBTEX. PMC9797047 36588793

[12] Hu H, Wang X, Zhang Y, Chen Q, Guan Q. A comprehensive survey on contrastive learning. Neurocomputing. 2024;610:128645. 10.1016/J.NEUCOM.2024.128645.

[13] Chen T, Kornblith S, Norouzi M, Hinton G. A simple framework for contrastive learning of visual representations. 37th International Conference on Machine Learning, ICML 2020 PartF168147-3: 1575–1585 (2020).

[14] Khosla P, Teterwak P, Wang C, Sarna A, Research G, Tian Y, et al. 2020. Supervised contrastive learning. Adv neural inform process syst. 33: 18661-18673.

[15] ENCODE Project Consortium: ENCDO776UMN: Human donor (25 years, female, European) metadata. https://www.encodeproject.org/human-donors/ENCDO776UMN/. Accessed: 2026-02-05 (2026).

[16] ENCODE Project Consortium: ENCDO832GKP: Human donor (31 years, male, healthy) metadata. https://www.encodeproject.org/human-donors/ENCDO832GKP/. Accessed: 2026-02-05 (2026).

[17] National Center for Biotechnology Information (NCBI) Gene Expression Omnibus: GSE76344: Histone modification and nucleosome mapping in human liver cancer cells. https://www.ncbi.nlm.nih.gov/geo/query/acc.cgi?acc=GSE76344. Accessed: 2026-02-05 (2020).

[18] ENCODE Project Consortium: ENCSR134DWG: Reference epigenome collection (HepG2 histone/chromatin data) metadata. https://www.encodeproject.org/reference-epigenomes/ENCSR134DWG/. Accessed: 2026-02-05 (2026).

[19] Primary data processing and quality control — processed data from the NIH Roadmap Epigenomics Mapping Consortium. https://egg2.wustl.edu/roadmap/web_portal/processed_data.html. Accessed: 2025-07-01 (2015).

[20] Kundaje Lab: PhantomPeakQualTools: Quality Metrics for ChIP-seq and Related Sequencing Assays. Bioconda / Anaconda.org. Conda package on Bioconda; accessed 2026-02-05. https://anaconda.org/bioconda/phantompeakqualtools

[21] Munif A. munif/epi-thesis. https://github.com/munif/epi-thesis/. Accessed: 2025-11-15 (2025).

[22] Infrastructure and Platforms: KAYA research compute platform. The University of Western Australia. Accessed: 2026–02-05 (2025). 10.26182/DH5S-V482

